# Stabilizing black phosphorus nanosheets via edge-selective bonding of sacrificial C_60_ molecules

**DOI:** 10.1038/s41467-018-06437-1

**Published:** 2018-10-09

**Authors:** Xianjun Zhu, Taiming Zhang, Daochuan Jiang, Hengli Duan, Zijun Sun, Mengmeng Zhang, Hongchang Jin, Runnan Guan, Yajuan Liu, Muqing Chen, Hengxing Ji, Pingwu Du, Wensheng Yan, Shiqiang Wei, Yalin Lu, Shangfeng Yang

**Affiliations:** 1 Hefei National Laboratory for Physical Sciences at Microscale, CAS Key Laboratory of Materials for Energy Conversion, Department of Materials Science and Engineering, Synergetic Innovation Center of Quantum Information & Quantum Physics, University of Science and Technology of China, Hefei, 230026 China; 2 CAS Key Laboratory of Materials for Energy Conversion, iChEM (Collaborative Innovation Center of Chemistry for Energy Materials), Department of Materials Science and Engineering, University of Science and Technology of China, Hefei, 230026 China; 3 National Synchrotron Radiation Laboratory, University of Science and Technology of China, Hefei, 230029 China

## Abstract

Few-layer black phosphorus (BP) with an anisotropic two-dimensional (2D)-layered structure shows potential applications in photoelectric conversion and photocatalysis, but is easily oxidized under ambient condition preferentially at its edge sites. Improving the ambient stability of BP nanosheets has been fulfilled by chemical functionalization, however this functionalization is typically non-selective. Here we show that edge-selective functionalization of BP nanosheets by covalently bonding stable C_60_ molecules leads to its significant stability improvement. Owing to the high stability of the hydrophobic C_60_ molecule, C_60_ functions as a sacrificial shield and effectively protects BP nanosheets from oxidation under ambient condition. C_60_ bonding leads to a rapid photoinduced electron transfer from BP to C_60_, affording enhanced photoelectrochemical and photocatalytic activities. The selective passivation of the reactive edge sites of BP nanosheets by sacrificial C_60_ molecules paves the way toward ambient processing and applications of BP.

## Abstract (web summary)

Few-layered black phosphorus has unique and appealing electronic properties but is easily oxidized in air. Here, the authors covalently functionalize black phosphorus nanosheets with C_60_ at their edges, providing improved stability and enhanced photoelectrochemical and photocatalytic activities.

## Introduction

Few-layer black phosphorus (BP), a two-dimensional (2D)-layered semiconductor that can be exfoliated from bulk BP, has been attracting increasing interest owing to its unique band structure featuring direct band gap with thickness-dependent gap energy in the range of 0.3–2.0 eV and high charge carrier mobility of ∼1000 cm^2^ V^−1^ s^−1^, rendering its potential applications in transistors, biomedicine, energy conversion, and storage^1–6^. Realization of such applications of few-layer BP is largely dependent on exfoliated BP nanosheets, which are however easily oxidized under ambient condition because each phosphorus atom has a pair of lone electrons prone to react with oxygen adsorbed on the surface of BP nanosheets^7–10^. The oxidation rate of BP nanosheets was found by Favron et al. to depend linearly on oxygen concentration dissolved in adsorbed water and light intensity^8^. A more recent report by Kuntz et al. revealed that pure oxygen led to oxidation of the van der Waals surface of BP, whereas water oxidized BP at pre-existing defects such as edges or steps^10^. Thus, improving the ambient stability of BP is a prerequisite for its practical applications^11^. So far several strategies have been developed to improve the ambient stability of BP nanosheets, which includes, for instance, protective layers coating^12,13^, heteroatoms doping^14^, and hybridization with other chemicals^15–18^ and chemical functionalization^19,20^. Among them, chemical functionalization has been implemented as one of the most effective routes to passivate the reactive BP, fulfilled by either covalent functionalization^19^, coordination^20^, or noncovalent functionalization via van der Waals (vdW) heterostructure formation or electrostatic interactions^21–23^. Noteworthy, these reported chemical functionalization approaches are typically non-selective, mostly on the surface of BP nanosheets^19–23^. Although surface functionalization can effectively passivate the phosphorus atoms located on the puckered surface, the involvement of functional groups can result in strong structure perturbation, consequently compromising the unique electronic structures of BP. Such an influence is amplified in few-layer BP nanosheets due to its improved specific surface area. On the other hand, P atoms at the edge sites are found to be more reactive in oxidation than those at the surface of phosphorene^10^. Thus, selective functionalization of BP, especially at its edges, is highly desired for passivating BP without losing its surface integrity. However, edge-selective functionalization of BP nanosheets has rarely been reported because of the difficulty in selectively activating its edge sites and finding a functional molecule with suitable reactivity which affords reaction with the more reactive edge sites only.

Fullerenes such as C_60_ as the first member of nanocarbon family are one of the most representative molecular materials, showing high stability against light, oxygen and water due to its hydrophobicity and spherical aromaticity^24–28^. These features along with its strong electron-accepting ability render the potential applications of fullerenes in versatile fields including energy conversion, catalysis, biomedicines^29–31^. Given that the low ambient stability of BP nanosheets is primarily due to its facile oxidation under the conditions of light, oxygen and water^7–10^, an intriguing idea is whether incorporating stable C_60_ molecules would prevent BP from oxidation. Besides, compared to such small molecules as aryl diazonium which afforded surface functionalization of BP^19^, the lower reactivity of C_60_ may benefit edge-selective functionalization of BP nanosheets. Nevertheless, to the best of our knowledge, up to now hybrid of BP and C_60_ has been scarcely reported primarily due to the difficulty on hybridization owing to their divergence on the dimensions.

Herein, we report the edge-selective bonding of C_60_ molecules onto BP nanosheets via covalent phosphorus–carbon bonds, accomplished via a facile one-step solid-state mechanochemical route by ball-milling bulk BP and C_60_ powders without any additive. On the basis of a series of morphological and spectroscopic characterizations, the successful edge-selective bonding of C_60_ within BP-C_60_ hybrid was confirmed. The effect of C_60_ bonding on the stability of BP nanosheets in water is studied. C_60_ molecules bonding at the edges of BP nanosheets serve as sacrificial shield, resulting in significant stability improvement of BP nanosheets against oxidation as well as obviously enhanced photoelectrochemical and photocatalytic activities of BP. Thus, our strategy on selectively passivating the reactive edge sites of BP nanosheets by sacrificial C_60_ molecules opens up new avenues for versatile applications of BP.

## Results

### Synthesis and characterization of the BP-C_60_ hybrid

**Fig. 1:**
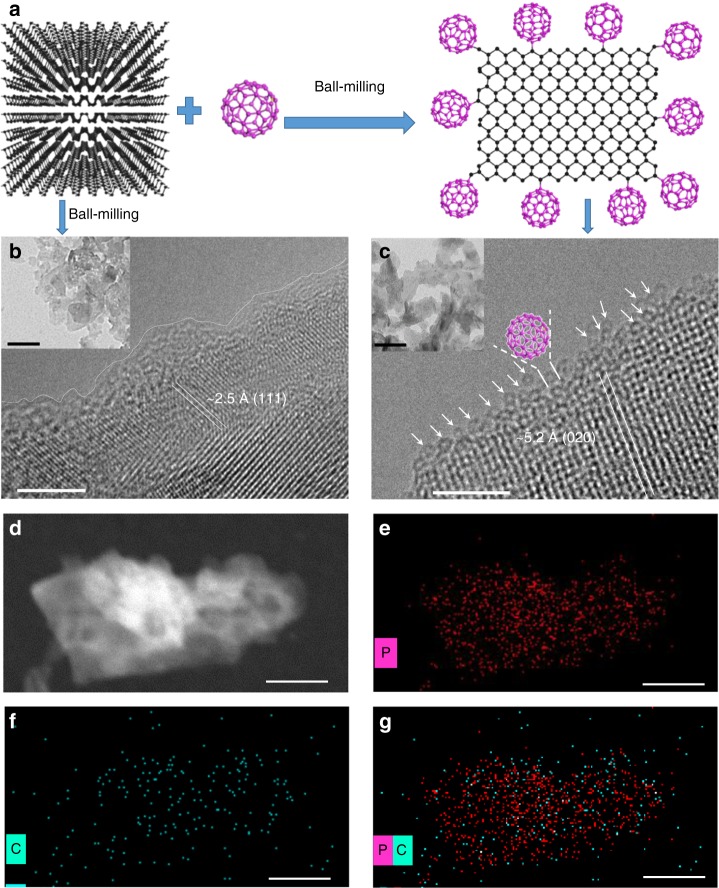
Microstructural characterizations. **a** Scheme of the preparation process and structure of BP-C_60_ hybrid. **b** HRTEM and low-magnification TEM (inset, scale bar: 100 nm) images of ball-milled BP (BP-BM). Inset: **c** HRTEM and low-magnification TEM (inset, scale bar: 100 nm) images of the BP-C_60_ hybrid. The arrows mark the C_60_ molecules. **d**–**g** STEM and EDX elemental (P and C) mapping images of the BP-C_60_ hybrid. The scale bars are 5, 5, 100, 100, 100, and 100 nm in **b**, **c**, **d**, **e**, **f**, and **g**, respectively

As an alternative route to the solution-phase chemical functionalization, solid-state mechanochemical method has recently been extensively applied to prepare edge-selectively functionalized graphene nanosheets by attaching different elements, small inorganic molecules or C_60_^32–34^. Given that BP has a 2D-layered structure analogous to graphene, we are stimulated to employ solid-state mechanochemical method to synthesize BP nanosheets with edge-selectively bonded C_60_ based on its simplicity and environmental-friendliness (without using any organic solvent). Three hundred milligrams of bulk BP, which was prepared by a phase transformation reaction from red phosphorus^35^, and 600 mg C_60_ powder were mixed and ball-milled directly under an Ar atmosphere in a planetary ball-milling machine (Fig. 1a). After ball-milling at 250 rpm for 24 h, the resultant black mixture was collected and Soxhlet-extracted with CS_2_ for 48 h to remove the unreacted C_60_. Note that, previously Cui et al. made an attempt to synthesize a BP-C_60_ composite via mechanochemical ball-milling route as a comparative study with BP-graphite composite synthesized under the same condition. However, in their report carbon materials including graphite and C_60_ were ball-milled prior to blending with BP, thus a two-step ball-milling process was needed for synthesizing their BP-C_60_ composite. Besides, whether or not C_60_ molecules bonded directly with BP remained unclear^15^. Hence, in our present study, we managed to simplify the process to one-step and unravel clearly how C_60_ molecules bond with BP.

Scanning electron microscopy (SEM) was carried out to study the morphology of BP-C_60_ hybrid. Compared to the bulk BP which shows large sheets with sizes of more than 10 μm (Supplementary Fig. 1a), BP-C_60_ hybrid exhibits aggregated particles with sizes of ∼1 μm in average (Supplementary Fig. 1b). For comparison, we also prepared two control samples obtained by ball-milling BP with LiOH as additive (abbreviated as BP-BM)^6^ and ball-milling C_60_ only (abbreviated as C_60_-BM) under the same ball-milling condition, and found that BP-BM shows smaller aggregated particles (Supplementary Fig. 1c) compared to that of BP-C_60_ hybrid. This is understandable since the existence of C_60_ would dissipate the mechanical energy of ball-milling effective for cleaving BP sheets. The average thickness of BP-C_60_ hybrid, acquired by atomic force microscopic (AFM) analyses (Supplementary Fig. 2), is ~2.5 nm, which is comparable to that of BP-BM (~2.7 nm) and corresponds to ~4-layer nanosheets based on the interlayer distance of ~0.52 nm (ref. ^2^). An average C_60_ molar content of 19 per 1000 P atoms was estimated according to the weight ratio of C_60_ (~30%) within the BP-C_60_ hybrid determined by thermogravimetric analysis (TGA) (Supplementary Fig. 3). The X-ray diffraction (XRD) pattern of BP-C_60_ hybrid shows intensive peaks at 16.8°, 34.2°, and 52.3°, which are indexed as the (020), (040), and (060) planes of BP, respectively (Supplementary Fig. 4), thus the crystal structure of BP is preserved after ball-milling. Besides, the signals of C_60_ crystals are invisible in the XRD pattern of BP-C_60_ hybrid, indicating the complete removal of unreacted C_60_ by Soxhlet-extraction.

The transmission electron microscopy (TEM) image of BP-BM shows flat nanosheets (inset of Fig. 1b, see also Supplementary Fig. 5b for a large-area image). According to the high-resolution TEM (HRTEM) image, the lattice fringes of (111) plane with space distance of ∼2.5 Å were observed at the edge of BP nanosheets, which is covered with amorphous coating due to the existence of defects resulted from P atom reconstruction at the edges of BP nanosheets during ball-milling process as well as the attachment of the hydroxyl groups at the edges introduced by LiOH additive (Fig. 1b). The amorphous coating may raise from oxidation of BP since the specimen had to be exposed in air for hours before transferred into TEM chamber. In contrast, the TEM image of the BP-C_60_ hybrid shows lattice fringes of (020) plane at the edges of the BP nanosheets without amorphous coating (Fig. 1c), even though the BP-C_60_ sample was exposed in air together with BP-BM, indicating that the BP-C_60_ hybrid has an improved structure stability at ambient condition. Interestingly, along the edges of the BP nanosheets, hollow nanospheres with diameter of ∼1.0 nm are observed in the high-resolution TEM (HRTEM) image of the BP-C_60_ hybrid (Fig. 1c), which match well with the morphology of C_60_ molecules with a van der Waals diameter of ∼1.0 nm^24,25^. These results indicate that C_60_ molecules primarily attach at the edges of BP nanosheets. Moreover, we carried out scanning transmission electron microscopy-energy dispersive X-ray spectroscopic (STEM-EDX) measurements to study the chemical distribution of the BP-C_60_ hybrid, and found that the P elements distribute homogeneously over the entire nanosheet whereas C elements have a much lower content than P elements (Fig. 1d–g). Thus, C_60_ bonding onto the surface of BP nanosheets can be excluded otherwise a much higher content of C elements should have been observed.

**Fig. 2:**
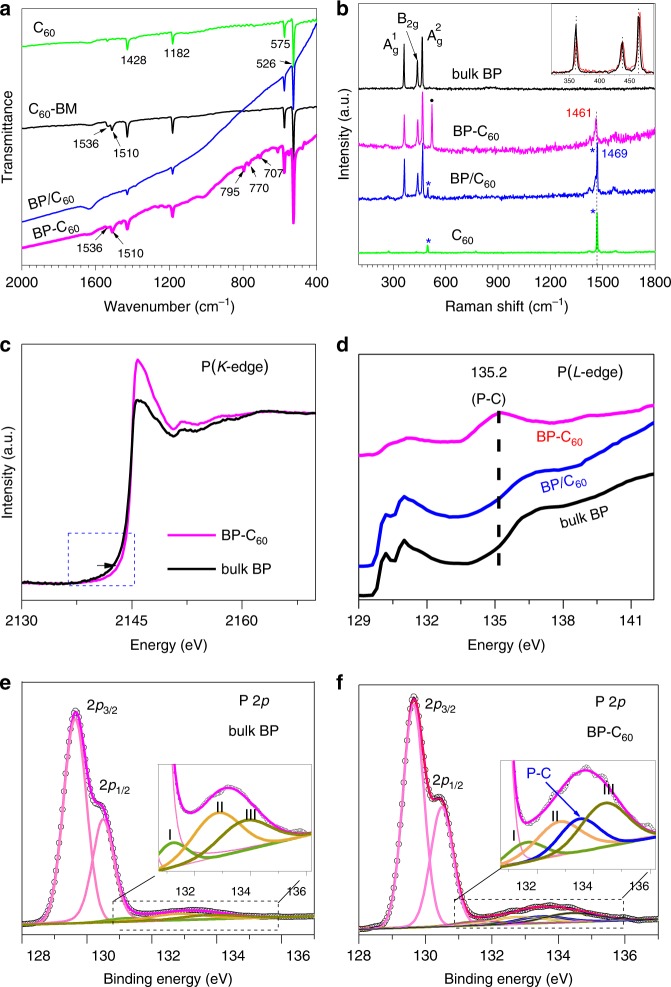
Spectroscopic characterizations. **a** FTIR spectra of the pristine C_60_, C_60_-BM, BP/C_60_ mixture and the BP-C_60_ hybrid. **b** Raman spectra of bulk BP, the BP-C_60_ hybrid, BP/C_60_ mixture, and pristine C_60_. The filled circle marks the signal of Si substrate, and the asterisks label the signal of C_60_. Inset: enlarged spectra of bulk BP and the BP-C_60_ hybrid. **c** P *K*-edge XAS spectra of bulk BP and the BP-C_60_ hybrid. **d** P *L*-edge XAS spectra of bulk BP, BP/C_60_ mixture and the BP-C_60_ hybrid. **e** High-resolution P2p XPS spectra of bulk BP. **f** High-resolution P2p XPS spectra of the BP-C_60_ hybrid. Numbers I-III label the signals assigned to P-O-P (bridging bonding), O–P=O (dangling bonding), and P_2_O_5_, respectively^6^

It is intriguing to determine whether C_60_ molecules attach onto the edges of BP nanosheets via covalent bonding or physical adsorption. We performed a series of spectroscopic characterizations to address this issue. For comparison, we prepared a physical mixture of BP-BM and C_60_ (abbreviated as BP/C_60_ mixture, in which the weight ratio of C_60_ is ~30%, close to that within BP-C_60_ hybrid) as a control sample. We first performed Fourier-transform infrared (FTIR) and solid-state ^13^C and ^31^P nuclear magnetic resonance (NMR) spectroscopic studies so as to investigate the bonding structure of C_60_ in the BP-C_60_ hybrid. The FTIR spectrum of the BP-C_60_ hybrid (Fig. 2a) shows four characteristic vibrational peaks of C_60_ at 526, 576, 1182, and 1428 cm^−1^ without appreciable shift relative to the pristine C_60_, indicating the existence of C_60_ moiety in the BP-C_60_ hybrid (see also Supplementary Fig. 6 for comparison with bulk BP and BP-BM). Besides, several new vibrational peaks at 707, 770, 795, 1510, and 1536 cm^−1^ appear in the spectrum of the BP-C_60_ hybrid. Except the vibrational peaks at 1510 and 1536 cm^−1^ which are also detected in the spectrum of C_60_-BM, the three new vibrational peaks at 707, 770, and 795 cm^−1^ are exclusively observed in the spectrum of the BP-C_60_ hybrid but absent in that of BP/C_60_ mixture. These results confirm that the BP-C_60_ hybrid is not a physical mixture of BP-BM, and the new vibrational peaks should originate from the as-formed phosphorus-carbon (P–C) covalent bonds^15,36^. In the solid-state ^13^C-NMR spectrum of BP-C_60_ hybrid, intense signals in the region of 130–160 ppm are detected, which are typical for *sp*^2^-carbon of C_60_, and an additional weak peak at 75.75 ppm is observed, which is absent in the spectra of both BP/C_60_ mixture and pure C_60_ (Supplementary Fig. 7), confirming the formation of *sp*^3^-carbon on the C_60_ cage. On the other hand, according to the comparison of the solid-state ^31^P NMR spectra of the BP-C_60_ hybrid, bulk BP and BP/C_60_ mixture, the intense signal at 21.68 ppm observed in bulk BP is obviously broadened with the appearance of several shoulder peaks in the high field in the spectra of both the BP-C_60_ hybrid and BP/C_60_ mixture probably due to the decreased crystallinity and the covalent functionalization of BP caused by ball-milling^6,37^. Interestingly, the signal peak at 21.68 ppm observed in bulk BP shifts to 22.86 ppm in the spectrum of the BP-C_60_ hybrid (Supplementary Fig. 7), suggesting a deshielding effect with decrease of electron density of the nucleus of P atom. On the contrary, for BP/C_60_-mixture such a signal peak negatively shifts to 19.62 ppm due to the covalent bonding of the hydroxyl functional groups^6^. The dramatic difference between the spectra of the BP-C_60_ hybrid and BP/C_60_-mixture solidifies the conclusion that the BP-C_60_ hybrid is not the physical mixture of BP and C_60_.

Figure 2b compares Raman spectra of the BP-C_60_ hybrid, bulk BP, pristine C_60_, and BP/C_60_ mixture. Bulk BP exhibits three intense Raman signals at 361, 437, and 465 cm^−1^, which are assigned to ${\mathrm{A}}_{\mathrm{g}}^1,{\hskip2pt}{\mathrm{B2}}_{{\mathrm{g}}}$ and ${\mathrm{A}}_{\mathrm{g}}^2$ modes, respectively^38^. In the Raman spectrum of pristine C_60_, two intense signals are observed at 495 and 1469 cm^−1^ corresponding to ${\mathrm{A}}_{\mathrm{g}}^1\,{\mathrm{and}}\,{\mathrm{A}}_{\mathrm{g}}^2$ mode, respectively^39^. For the BP/C_60_ mixture, its Raman spectrum shows a superposition of signals of BP and C_60_ moieties without any shifts, indicating no strong intermolecular interactions between BP and C_60_ moieties. Instead, the Raman spectrum of the BP-C_60_ hybrid is apparently different from that of BP/C_60_-mixture in terms of the absence of the signal at 495 cm^−1^ (${\mathrm{A}}_{\mathrm{g}}^1$ mode) and the down-shift of the most intense ${\mathrm{A}}_{\mathrm{g}}^2$ mode (1469 cm^−1^) of pristine C_60_ by ∼8 to 1461 cm^−1^. Since the latter peak (${\mathrm{A}}_{\mathrm{g}}^2$ mode) is sensitive to the charge state of C_60_, such a down-shift suggests that charge transfer from BP to C_60_ may take place due to the well-known strong electron-accepting ability of C_60_^24,34^. These results indicate that the BP-C_60_ hybrid is not a physical mixture of BP-BM and C_60_ otherwise charge transfer from BP to C_60_ could not have happened, instead C_60_ molecules attach onto the edges of BP nanosheets via covalent bonding. Besides, the three characteristic Raman peaks of bulk BP show negligible shifts and relative intensity change in the spectrum of the BP-C_60_ hybrid (see inset of Fig. 2b), confirming that C_60_ molecules primarily attach at the edges of BP nanosheets otherwise obvious change of their relative intensity would have been visible if surface grafting of C_60_ molecules onto BP nanosheets occurred^19^.

To further analyze the bonding structure of BP in the BP-C_60_ hybrid, we carried out X-ray absorption spectroscopic (XAS) and X-ray photoemission spectroscopic (XPS) studies. P *K*-edge XAS spectra of the BP-C_60_ hybrid and bulk BP are compared in Fig. 2c. The XAS spectrum of the BP-C_60_ hybrid exhibits a more intense peak at 2145.8 eV and a blue-shift by ∼1 eV of the absorption edge compared to that of bulk BP, indicating the electron transfer from BP to C_60_^40,41^. We further collected P *L-*edge XAS spectra of the BP-C_60_ hybrid and bulk BP, which provide richer spectral features than the *K*-edge spectra^42^. As shown in Fig. 2d, the P *L-*edge XAS spectrum of bulk BP exhibits near-edge absorption peaks in the region of 129.5–132.5 eV corresponding to the P(2p) → 1e* transition and a broad peak at around 137.0 eV ascribed to transitions from 2*p*_3/2_ and 2*p*_1/2_ levels^43,44^. However, in the P *L*-edge XAS spectrum of the BP-C_60_ hybrid, a new peak centered at ∼135.2 eV appears, which can be assigned to the as-formed P–C bonds^8,38^. Noteworthy, this peak is absent in the P *L*-edge XAS spectrum of the BP/C_60_ mixture, confirming further that the BP-C_60_ hybrid is not a physical mixture of BP-BM and C_60_.

Figure 2e, f compares the high-resolution P 2*p* spectra of the BP-C_60_ hybrid and bulk BP. In the high-resolution P 2*p* XPS spectrum of bulk BP, two intense peaks centered at 129.6 and 130.5 eV are attributed to 2*p*_3/2_ and 2*p*_1/2_ of P–P bonds, while a weak broad peak at ∼133.0 eV is due to the unavoidable oxidation of BP (Fig. 2e)^6,8,45–47^. For the BP-C_60_ hybrid, although the overall P 2*p* XPS spectrum looks quite similar (Supplementary Fig. 8), a detailed deconvolution analysis reveals the appearance of a new peak centered at ∼133.5 eV, which can be assigned to P-C covalent bonds (Fig. 2f)^19^.

### Formation mechanism of the BP-C_60_ hybrid

Based on the aforementioned characterizations, we propose a plausible conformation of the BP-C_60_ hybrid as well as its formation mechanism as illustrated in Supplementary Fig. 9. Similar to the studies of mechanochemical functionalization of graphene extensively reported in the literatures^32–34^, high-energy ball-milling of bulk BP results in its exfoliation to few-layer BP nanosheets along with the generation of the reactive species (radicals and ions) at the edges via a mechanochemical cleavage of P–P bonds. Simultaneously, C_60_ is activated by high-energy ball-milling^33^, and consequently attaches onto the activated edges of BP nanosheets via the covalent P-C bonds. In this sense, the attachment of C_60_ onto the surface of BP nanosheets is less preferable because the average thickness of the BP-C_60_ hybrid (2.5 ± 0.2 nm) is even slightly smaller than that of BP-BM (2.7 ± 0.2 nm) according to AFM analyses (Supplementary Fig. 2). Thus, bonding of C_60_ onto BP nanosheets is highly edge-selective. Noteworthy, such an edge-selective bonding of C_60_ onto BP nanosheets can be accomplished by blending bulk BP and C_60_ directly without any additive, thus being very facile and eco-friendly. In addition, we also carried out a control experiment with an attempt to improve the yield of the BP-C_60_ hybrid by ball-milling the mixture of bulk BP and C_60_ in the existence of LiOH, which was a crucial additive for the formation of graphene-C_60_ hybrid as reported previously^34^, but surprisingly found almost no hybrid formation according to Raman spectroscopic analysis (Supplementary Fig. 10).

### Ambient stability of the BP-C_60_ hybrid

**Fig. 3:**
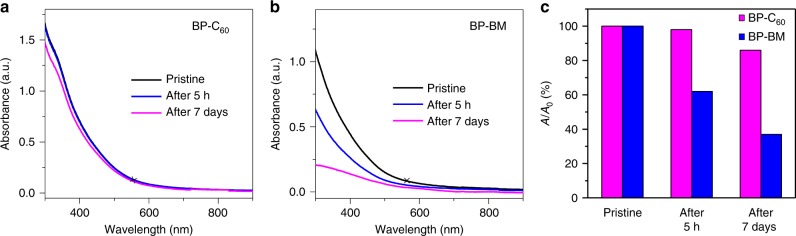
Stabilities of the BP-C_60_ hybrid and BP-BM dispersions in water. UV-vis absorption spectra of the BP-C_60_ hybrid (**a**) and BP-BM (**b**) dispersed in water after standing for different times. **c** Variation of the absorption ratios (*A*/*A*_0_) at 550 nm (marked by a cross) of BP-C_60_ and BP-BM dispersions with different times

It is well known that BP nanosheets can be easily oxidized under ambient condition, and previous reports reveal that surface functionalization of BP can effectively passivate the P atoms located on the puckered surface and consequently stabilize BP nanosheets^19,20^. It is thus stimulating to investigate whether the edge-selective functionalization by C_60_ can stabilize BP nanosheets as well. We monitored the optical absorbances of BP-C_60_ hybrid and BP-BM which are both dispersed in water under ambient condition^20^. In the UV-vis absorption spectrum of the BP-C_60_ hybrid dispersion, a shoulder peak at ∼340 nm appears (Fig. 3a), which is absent in the spectrum of BP-BM dispersion (Fig. 3b), confirming the existence of C_60_ moiety within the BP-C_60_ hybrid (Supplementary Fig. 11). Noteworthy, the UV-vis absorbance of BP-C_60_ dispersion at 550 nm shows only a slight decrease (by ∼2%) after standing for 5 h, whereas there is a ∼38% decrease in the absorbance for BP-BM dispersion. After 7 days, the absorbance of BP-C_60_ dispersion retains at ∼86%, which is much larger than that of BP-BM dispersion (∼36%, Fig. 3c). Therefore, the degradation rate of BP-C_60_ in water is significantly inhibited by a factor of 4.6 compared to that of BP-BM. The significant improvement of the stability of the BP-C_60_ hybrid is attributed to the bonding of stable C_60_ molecules via the covalent P-C bonds as confirmed by the above spectroscopic studies. Since C_60_ molecules have high stability against light, oxygen, and water^24–27^, bonding of C_60_ onto the edges of BP nanosheets provides a sacrificial shield which effectively prevents BP from attacks of light, oxygen and water (see Supplementary Fig. 9)^10^. Thus, we demonstrate that edge-selective functionalization by C_60_ can stabilize BP nanosheets other than surface functionalization.

### Photoelectrochemical and photocatalytic activities

**Fig. 4:**
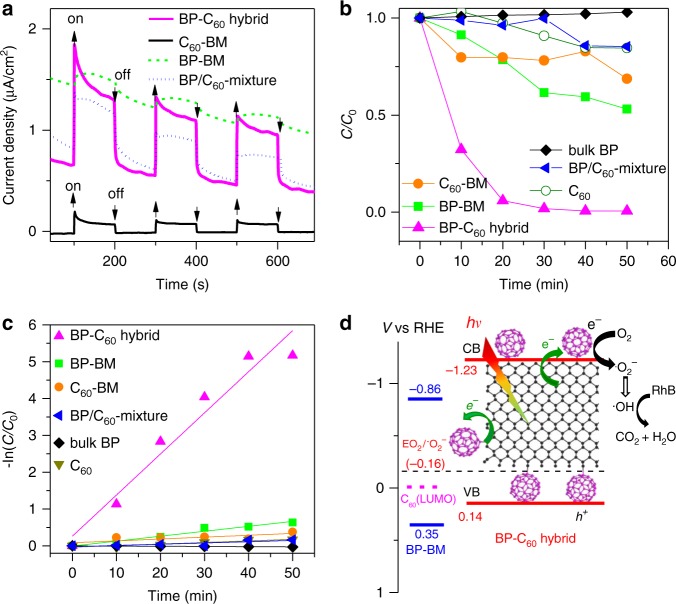
Photocurrent response and photocatalytic activities. **a** Photocurrent-time dependences of the BP-C_60_ hybrid, BP/C_60_ mixture, BP-BM, and C_60_-BM. For bulk BP, no photocurrent response was detected since the VB level of bulk BP (−0.01 V vs RHE)^6^ is even more negative than the work function of FTO, leading to a facile recombination of the photogenerated electron–hole pairs. **b** Photocatalytic degradation of RhB over the BP-C_60_ hybrid, BP/C_60_ mixture, BP-BM, C_60_-BM, pristine C_60_ and bulk BP powders under visible light. *C* and *C*_0_ denote the reaction and absorption equilibrium concentrations of RhB in the system. **c** Pseudo-first-order kinetics curves of RhB degradation over different samples. **d** The experimentally determined energy levels of the BP-C_60_ hybrid and BP-BM, and a schematic illustration showing the mechanism of the photocatalytic dye degradation of the BP-C_60_ hybrid. The lowest unoccupied molecular orbital (LUMO) energy level of C_60_ is also shown for comparison (see caption of Supplementary Fig. 13 for details)

Upon improving the stability of BP nanosheets in water via edge-selective bonding of C_60_, we next applied the BP-C_60_ hybrid in photoelectrochemical cell and photocatalytic dye degration. Figure 4a shows a typical photocurrent response curve of a photoelectrochemical cell composed of a BP-C_60_ hybrid-modified FTO transparent electrode measured at a bias voltage of 0.2 V. Clearly, a photocurrent switching during light on–off cycles is observed, and the net photocurrent induced by the initial light illumination is ∼1.2 μA cm^−2^, which attenuates rapidly during the light illumination of 100 s (Fig. 4a). Such a photocurrent overshooting phenomenon upon light illumination has been often reported for photoelectrochemical cells based on semiconducting inorganic nanostructures, and is presumably due to the back electron transfer process^48^. For comparison, we also measured the photocurrent responses of BP–BM and C_60_-BM under the identical conditions. BP-BM exhibits a photocurrent response of ∼0.12 μA cm^−2^, which is comparable to that of BP nanosheets with comparable thickness reported in ref. ^49^. The photocurrent of the BP-C_60_ hybrid is around 10 times of that of BP-BM and also much higher than that of C_60_-BM (∼0.20 μA cm^−2^), indicating that the hybrid conformation is advantageous for enhancing the photoelectric conversion properties of both BP and C_60_. Compared to BP/C_60_ mixture for which a photocurrent response of ∼0.57 μA.cm^−2^ is obtained, the photocurrent response of the BP-C_60_ hybrid is around two times higher. This indicates again that BP-C_60_ hybrid and BP/C_60_ mixture are quite different, and the covalent bonding of C_60_ onto BP is crucial for improving the photocurrent response. Since the optical absorption edge of the BP-C_60_ hybrid is comparable to that of BP-BM (Supplementary Fig. 12a), the enhanced photoelectric conversion property can be attributed to the photoinduced electron transfer from BP to C_60_ within the BP-C_60_ hybrid as revealed by steady-state photoluminescence (PL) spectroscopic study (Supplementary Fig. 13a), which takes place in the order of tens to hundreds of picoseconds according to time-resolved photoluminescence (TRPL) spectroscopic result (Supplementary Fig. 13b).

We further evaluated the effect of C_60_ bonding on the photocatalytic activity of BP for degradation of Rhodamine B (RhB) dye under visible light. Figure 4b compares the photocatalytic activities of the BP-C_60_ hybrid, BP/C_60_ mixture, bulk BP, pristine C_60_, BP-BM, and C_60_-BM. While bulk BP, pristine C_60_, BP-BM, C_60_-BM, and BP/C_60_ mixture all show low photocatalytic activity, the BP-C_60_ hybrid exhibits significantly enhanced photocatalytic activity with an apparent reaction rate constant (*k*) of 6.70 h^−1^ based on fitting to a pseudo-first-order kinetics (Fig. 4b, c)^50^. Noteworthy, the photocatalytic activity of BP/C_60_ mixture is much lower than that of the BP-C_60_ hybrid, and is even lower than that of BP-BM due mainly to the decrease of the content of BP-BM (~70 wt%). This result reveals the advantage of the BP-C_60_ hybrid formation in enhancing the photocatalytic activity of BP. To evaluate the stability of the BP-C_60_ hybrid during photocatalytic degradation reactions, we measured the TEM image and P 2*p* XPS spectrum of the BP-C_60_ hybrid after photocatalytic degradation reactions (Supplementary Figs. 14, 15), and found that the BP-C_60_ hybrid kept the original form of nanosheets with comparable sizes to the original one, and no obvious oxidization occurred. Thus, the high stability of the BP-C_60_ hybrid against oxidation during the photocatalytic degradation reactions is confirmed.

To unravel the mechanism responsible for the enhanced photocatalytic activity of the BP-C_60_ hybrid, we measured its conduction band (CB) and valence band (VB) energy levels by synchrotron radiation photoemission spectroscopy (SR-PES), which are −3.27 and −4.64 eV versus vacuum level (−1.23 and 0.14 V vs reversible hydrogen electrode (RHE)), respectively (Supplementary Figs. 16, 17 and Supplementary Table 1). Compared to BP-BM with CB/VB levels of −0.86/0.35 V vs RHE, both of the CB and VB levels of the BP-C_60_ hybrid shift negatively. In particular, the more negative CB level of the BP-C_60_ hybrid facilitates the reduction of the dissolved O_2_ to generate ^●^O_2_^−^(ref. ^46^.), which then transforms to ^●^OH radicals^50^ and consequently degrade RhB (Fig. 4d). Indeed, the gradual generation of .OH radicals during the photocatalytic process was confirmed by monitoring the formation of hydroxyl radicals over the visible light irradiation period of 40 min by PL spectroscopy (Supplementary Fig. 18).

## Discussion

In summary, we report edge-selective functionalization of BP nanosheets by covalently bonding stable C_60_ molecules, leading to significant stability improvement of BP owing to the sacrificial C_60_ shield. The edge-selective bonding of C_60_ molecules via covalent P–C bonds was accomplished via a one-step ball-milling of the BP and C_60_ mixture without any additive, thus being very facile and eco-friendly. Owing to the high stability of the hydrophobic C_60_ molecules against light, oxygen, and water, C_60_ effectively protects BP nanosheets from oxidation, thus significantly improves the stability of BP nanosheets in water with the degradation rate inhibited by a factor of 4.6. The involvement of C_60_ leads to the photoinduced electron transfer from BP to C_60_ within the BP-C_60_ hybrid, which can inhibit the recombination of charge carriers and consequently enhance not only the photoelectric conversion property but also the photocatalytic activity of BP. Our conceptual breakthrough on selectively passivating the reactive edge sites of BP nanosheets by sacrificial C_60_ molecules paves the way towards ambient processing and applications of BP.

## Methods

### Preparation of the BP-C_60_ hybrid

Bulk BP was synthesized by a phase transformation reaction from red phosphorus following a similar method reported in literature^35^. The BP-C_60_ hybrid was synthesized in a planetary ball-milling machine. Typically, a mixture of 300 mg bulk BP powder and 600 mg C_60_ powder was put into a ZrO_2_ ball-milling jar containing 50 g ZrO_2_ balls (3 mm diameter). The jar was sealed in a glovebox filled with Ar and finally equipped on the planetary ball-milling machine. The ball-milling process was performed at ambient temperature for total 24 h (with 15 min interval for every 30 min milling time) with the rotation speed of 250 rpm. After ball-milling, the resultant mixture was collected and Soxhlet-extracted with CS_2_ for 48 h to remove the unreacted C_60_. Finally, the sample was vacuum dried for 1 day at 40 °C.

The control sample BP-BM was prepared by ball-milling a mixture of 270 mg bulk BP powder and 630 mg anhydrous LiOH powder for 12 h with 50 g ZrO_2_ (3 mm diameter)^6^. C_60_-BM was synthesized by ball-milling pristine 1 g C_60_ powder for 12 h with 50 g ZrO_2_ (3 mm diameter), followed by Soxhlet-extraction with CS_2_ for 48 h to remove the unreacted C_60_. The BP/C_60_ mixture was prepared through stirring the BP-BM powders (70 wt%) and C_60_ powders (30 wt%) in the 10 mL isopropanol solution by 24 h and removing solvent by centrifugation.

### Stability measurements

The BP-C_60_ and BP-BM powders were dispersed in water and sonicated for 30 min, followed by 20 min centrifugation at 9000 rpm to get dispersed BP-C_60_ and BP-BM nanosheets aqueous solution. The BP-C_60_ and BP-BM nanosheet aqueous solutions were kept under ambient condition for different periods, and the optical absorbance at each time point was monitored so as to investigate their stability according to the change of the optical absorbance intensity.

### Photoelectrochemical measurements

The sample (15 mg) was dispersed in ethanol (300 μL), ultrasonicated for 30 min to form a homogenous ink. A volume of 100 μL of the ink was dropped onto the conductive side of FTO slide (1 cm × 2 cm) coating ~1 cm^2^, followed by vacuum drying. The films on FTO were used as working electrode. The electrochemical experiments were carried out on an electrochemical workstation (CHI 660C, Shanghai ChenHua Instrument Company, China). With a typical three-electrode cell under the ambient condition and a 300 W xenon lamp (Lansheng Electronic Co., China) as light source. The counter electrode and reference electrode were a platinum wire and a silver wire. The electrolyte was 0.1 M KCl aqueous solution.

### Photocatalytic measurements

A 5 mg sample was placed in 50 mL 0.01 mg mL^−1^ RhB solution in a 50 mL beaker. The solution was magnetically stirred in dark for 60 min to ensure the establishment of an adsorption/desorption equilibrium between the photocatalyst and organic pollutants. Photocatalytic degradation of RhB by the powder samples under visible light irradiation was performed using a 300 W xenon lamp with a UV cutoff filter (*λ* > 420 nm) as light. Before irradiation, 2 mL solution was collected and tested by using UV-vis-NIR spectrometer to measure the concentration of the solution after adsorption/desorption equilibrium. During irradiation, about 2 mL solution was collected and tested by using UV-vis-NIR spectrometer every 10 min. The concentration of RhB was measured according to the absorbance at 550 nm.

To detect the formation of hydroxyl radicals, 5 mg BP-C_60_ hybrid powder was placed in 50 mL terephthalic acid solution containing (4 × 10^−4^ M terephthalic acid and 2 × 10^−3^ M NaOH). The solution was irradiated by a 300 W halogen lamp (with a 400 nm filter). At every 10 min, 1.5 mL suspensions were collected and centrifuged. The resulted supernatants were subjected to PL measurements to detect the fluorescence spectra of the generated 2-hydroxyterephthalic acid.

### Characterization

FTIR spectra were performed on a TENSOR 27 spectrometer (Bruker, Germany) at room temperature. The Raman spectra were obtained at room temperature with an in Via Raman Microscope equipment (Renishaw, England) with a 532 nm excitation laser. Solid-state ^13^C nuclear magnetic resonance (NMR) spectra was carried out on a AVANCE AV400 magic angle spinning (MAS) measurements (Bruker, Germany) using a standard Bruker 4 mm MAS probe with spinning speed 14 kHz. SEM images were obtained from a JEOL JSM-6390LA instrument (Rigaku, Japan). The high-resolution transmission electron microscopy (HRTEM) was conducted on a JEOL-2010 (Rigaku, Japan) microscope operating at a voltage of 200 kV. The scanning transmission electron microscopy-energy dispersive X-ray (STEM-EDX) mapping images were obtained on a JEOL-2100F (Rigaku, Japan) microscope operating at a voltage of 200 kV. X-ray photoelectron spectroscopy (XPS) was conducted on a Thermo-VV ESCALAB 250 (Thermo-VV Scientific) machine. Atomic force microscopy (AFM) measurements were carried out on a XE7 scanning probe microscope (Park, Korea). XRD patterns were obtained from a Smart Lab 9 kW X-ray diffraction instrument (Rigaku, Japan). Thermal gravimetric analysis (TGA) analysis was carried out on a Q600 SDT instrument (TA, USA) under N_2_ atmosphere. UV-vis-NIR diffuse reflection spectroscopy (DRS) was performed on a 3700 UV-vis spectrometer (Shimadzu, Japan). The Carbon *K*-edge X-ray absorption spectroscopy (XAS) and phosphorus *L*_2,3_-edge XAS were collected with a 0.1 eV energy resolution at beamline 12B-α of the National Synchrotron Light Source (Heifei, China) at National Synchrotron Radiation Laboratory. The Phosphorus K-edge XAS were collected at beamline 16A1 of the National Synchrotron Radiation Research Center (Hsinchu, Taiwan, China). Synchrotron radiation photoemission spectroscopy (SR-PES) experiments were performed at the Catalysis and Surface Science endstation in the National Synchrotron Radiation Laboratory (NSRL), Hefei, and measured using synchrotron radiation light as the excitation source with a photon energy of 39.9 eV. A sample bias of −5 V was applied to observe the secondary electron cutoff. The fluorescence spectra were measured on a Hitachi F-4500 fluorescence spectrophotometer with an excitation wavelength of 320 nm. The steady-state photoluminescence (PL) spectra were measured using an fluorescence spectrometer (Jobin Yvon, France) with an excitation wavelength of 450 nm.

## Electronic supplementary material

Supplementary Information

## Data Availability

All data supporting the findings of this study are available from the corresponding author on request.
