# Supplementary material for: Stabilizing black phosphorus nanosheets via edge-selective bonding of sacrificial C60 molecules
Source: Nat Commun. 2018 Oct 9;9:4177. doi: 10.1038/s41467-018-06437-1 (PMC6177470; doi:10.1038/s41467-018-06437-1)
Supplement: Supplementary file 1 — Supplementary Information [file 41467_2018_6437_MOESM1_ESM.pdf]

SUPPLEMENTARY INFORMATION

Stabilizing black phosphorus nanosheets via edge-selective bonding of sacrificial C_{60} molecules

Xianjun Zhu et al.

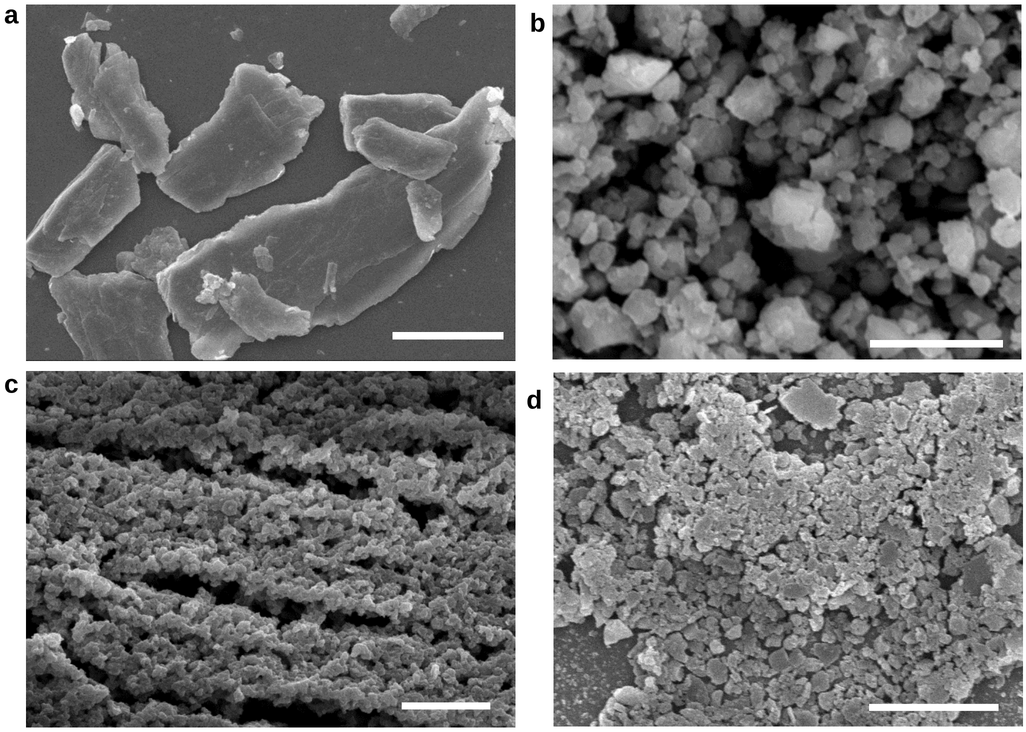

Supplementary Figure 1 | SEM images of bulk BP **(a),** the BP-C_{60} hybrid **(b),** bulk BP ball-milled in the existence of LiOH as additive (BP-BM, **c**) and pure C_{60} ball-milled (C_{60}-BM, **d**). The scale bars are 10 μm.

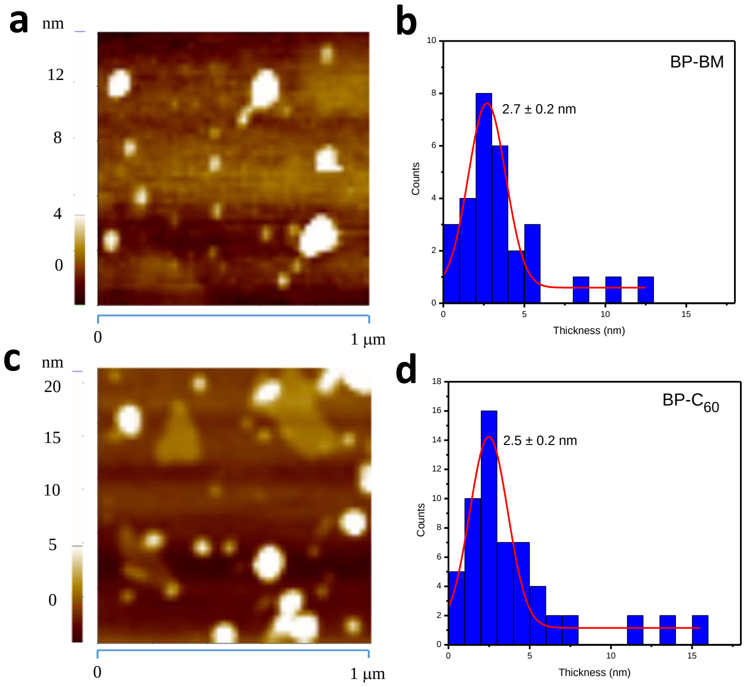

Supplementary Figure 2 | High-magnification AFM images (a, c) and the thickness distribution (b, d) of BP-BM and the BP-C_{60} hybrid.

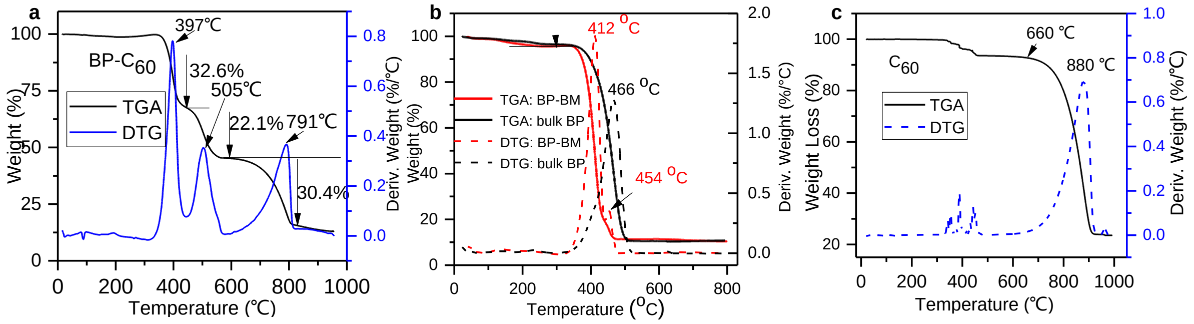

Supplementary Figure 3 | Thermogravimetric analysis (TGA) and derivative thermogravimetric (DTG) curves of the BP-C_{60} hybrid (a), bulk BP and BP-BM (b), and pristine C_{60} (c). According to TGA curve of BP-C_{60} hybrid, three weight loss processes were clearly observed for BP-C_{60} hybrid. In the first weight loss step (300 - 440 °C), a ~33% weight loss is observed, corresponding to the decomposition of BP (ref. 2). In the second weight loss process (440 - 572 °C), a ~22% weight loss can be assigned to the thermal detachment of C_{60} from BP and the following decomposition of BP. Interestingly, in this process the agravic peak at 505 °C detected in derivative thermogravimetric (DTG) curve of BP-C_{60} hybrid is higher than those of bulk BP (~466 °C) and BP-BM (~412/454 °C), indicating that the thermal stability of BP are enhanced due to the edge-selective attachment of C_{60}, which is thermally stable up to ~660 °C. Finally, a ~30% weight loss in the third weight loss process is assigned to the decomposition of C_{60}. According to the ratio of the C_{60} weight loss, an average C_{60} molar content is estimated to be 19 per 1000 within BP-C_{60} hybrid.

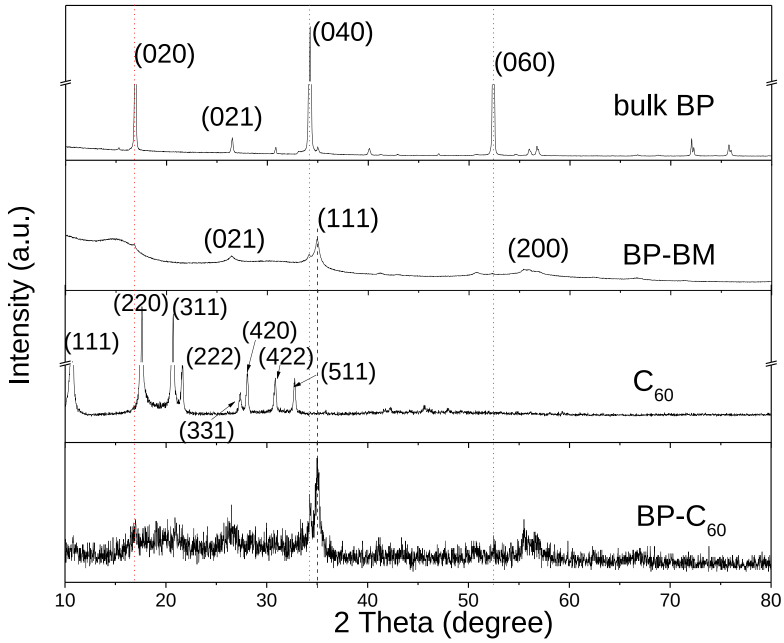

Supplementary Figure 4 | XRD pattern of the BP-C_{60} hybrid, bulk BP, BP-BM and pristine C_{60}. According to the comparison of the XRD patterns of the BP-C_{60} hybrid and bulk BP, the three intense diffraction peaks at 16.8°, 34.2° and 52.3°, which are indexed as the (020), (040) and (060) planes of BP respectively[1], keep un-shifted in BP-C_{60} hybrid, suggesting that the layer-to-layer d-spacing keeps constant, thus C_{60} intercalate into the space between layers of BP nanosheets can be ruled out otherwise such an intercalation may induce change on the layer-to-layer distance like the intercalated graphite. However, the characteristic diffraction peaks of bulk BP exhibit dramatic intensity decreases for both BP-C_{60} hybrid and BP-BM, suggesting that a high degree of exfoliation of BP layers occurs during the ball milling. Besides, no peak assigned to C_{60} crystals is observed in the XRD pattern of the BP-C_{60} hybrid, indicating that C_{60} molecules do not crystallize separately on the BP nanosheets.

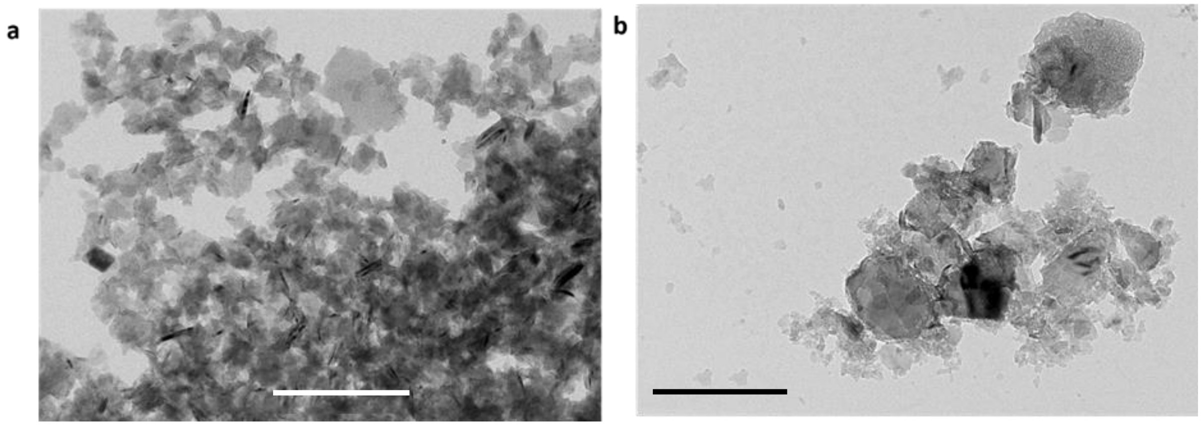

Supplementary Figure 5 | TEM images of the BP-C_{60} hybrids (a) and BP-BM (b). The scale bars are 500 nm.

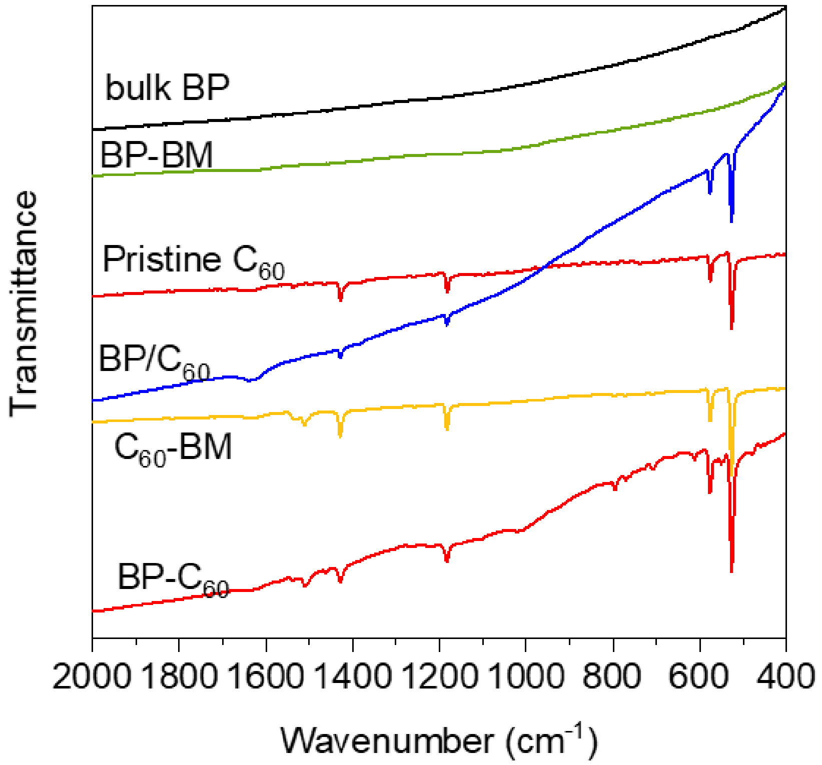

Supplementary Figure 6 | FTIR spectra of bulk BP, BP-BM, pristine C_{60}, C_{60}-BM, BP/C_{60} mixture and the BP-C_{60} hybrid.

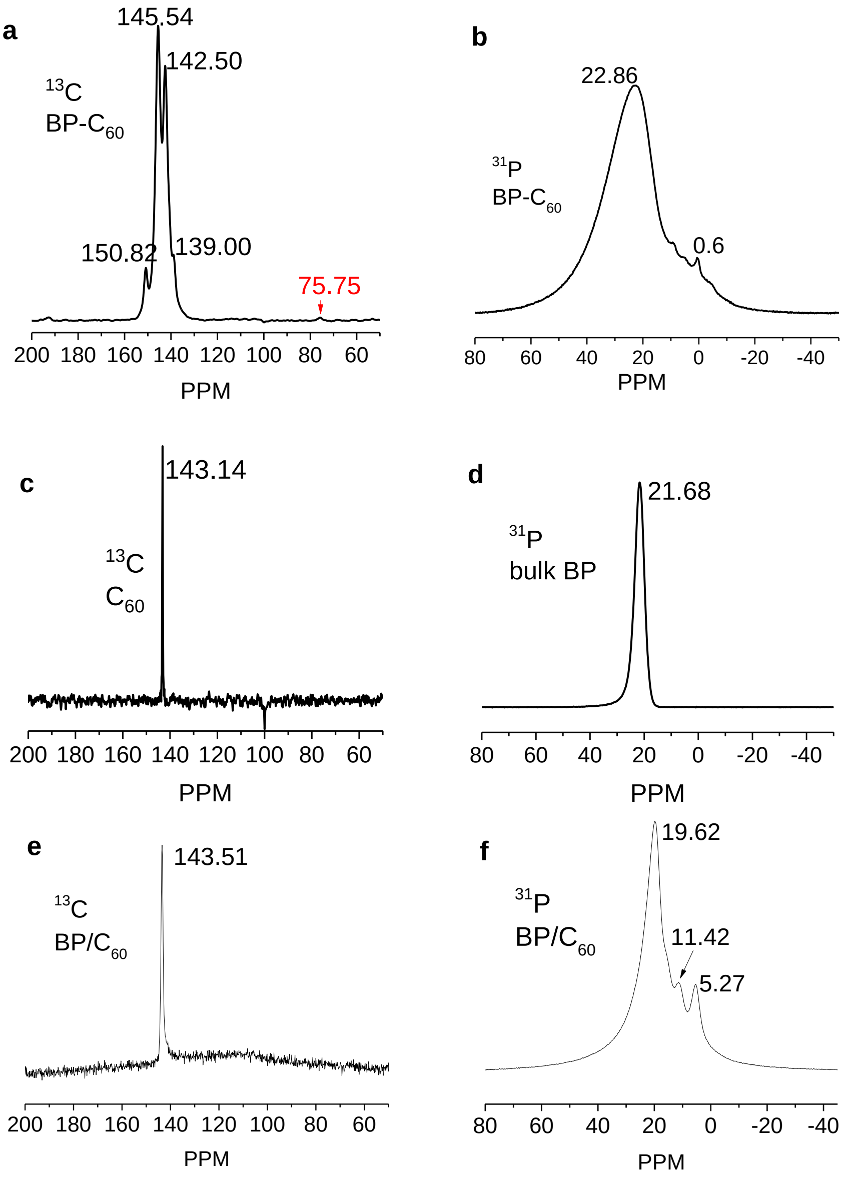

Supplementary Figure 7 | Solid-state ^{13}C nuclear magnetic resonance (NMR) spectra of the BP-C_{60} hybrid (**a**), pure C_{60} (**c**) and BP/C_{60}-mixture (**e**). The weak peak at 75.75 ppm in the spectrum of the BP-C_{60} hybrid corresponds to *sp*^{3}-carbon on the C_{60} cage. Solid-state ^{31}P NMR spectra of the BP-C_{60} hybrid (**b**), bulk BP (**d**) and BP/C_{60}-mixture (**f**). The intense signal at 21.68 ppm observed in bulk BP is obviously broadened with the appearance of several shoulder peaks in the high field in the spectra of both the BP-C_{60} hybrid and BP/C_{60}-mixture probably due to the decreased crystallinity and the covalent functionalization of BP caused by ball-milling[2]. Interestingly, the signal peak at 21.68 ppm observed in bulk BP shifts to 22.86 ppm in the spectrum of the BP-C_{60} hybrid, suggesting a deshielding effect with decrease of electron density of the nucleus of P atom. On the contrary, for BP/C_{60}-mixture such a signal peak negatively shifts to 19.62 ppm due to the covalent bonding of the hydroxyl functional groups[1,2]. The dramatic difference between the spectra of the BP-C_{60} hybrid and BP/C_{60}-mixture solidifies the conclusion that the BP-C_{60} hybrid is not the physical mixture of BP and C_{60}.

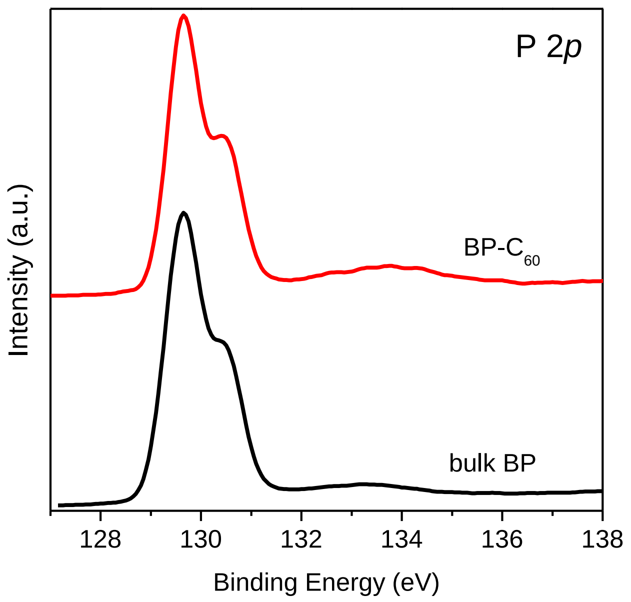

Supplementary Figure 8 | P 2*p* XPS spectra of the BP-C_{60} hybrid and bulk BP.

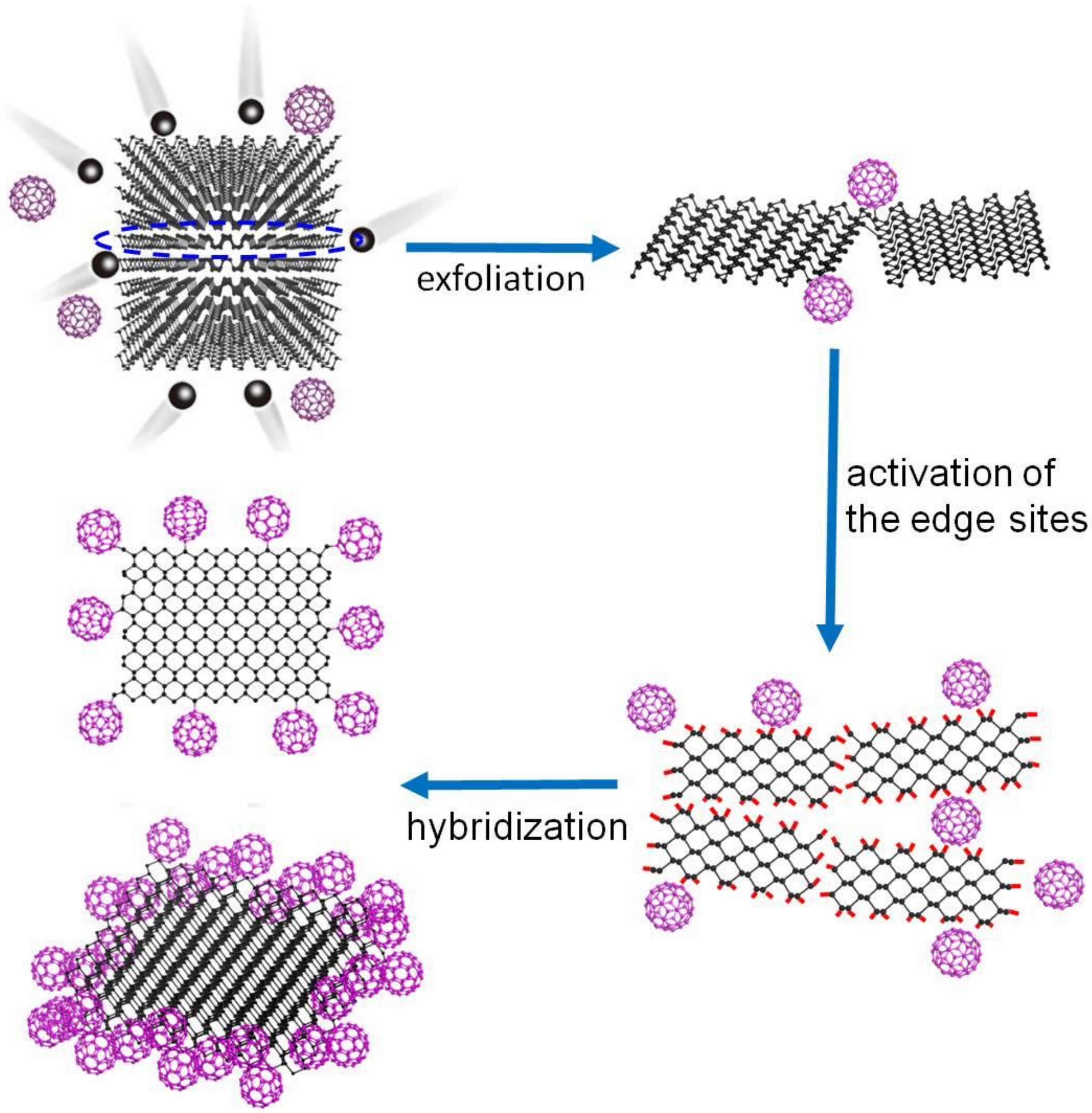

Supplementary Figure 9 | Schematic illustration of the mechanochemical reaction between BP and C_{60} during ball-milling process. The red short lines represent the active sites at edges of BP nanosheets generated during ball-milling. The as-formed BP-C_{60} hybrid was shown in two different views. Note that the sizes of milling balls, C_{60} molecules and BP nanosheets are not proportional to their actual sizes.

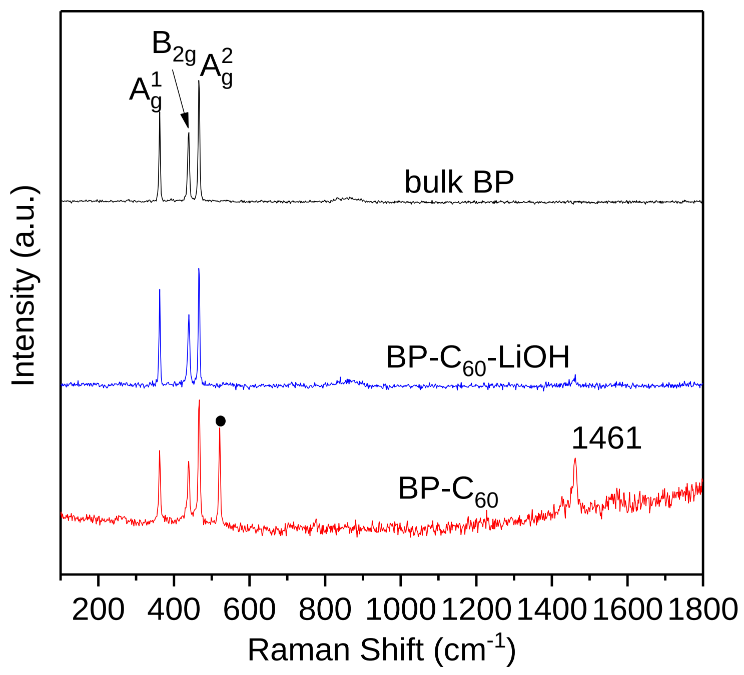

Supplementary Figure 10 | Raman spectra of bulk BP and mixture of BP and C_{60} ball-milled with and without LiOH (The filled circle labels Si signal).

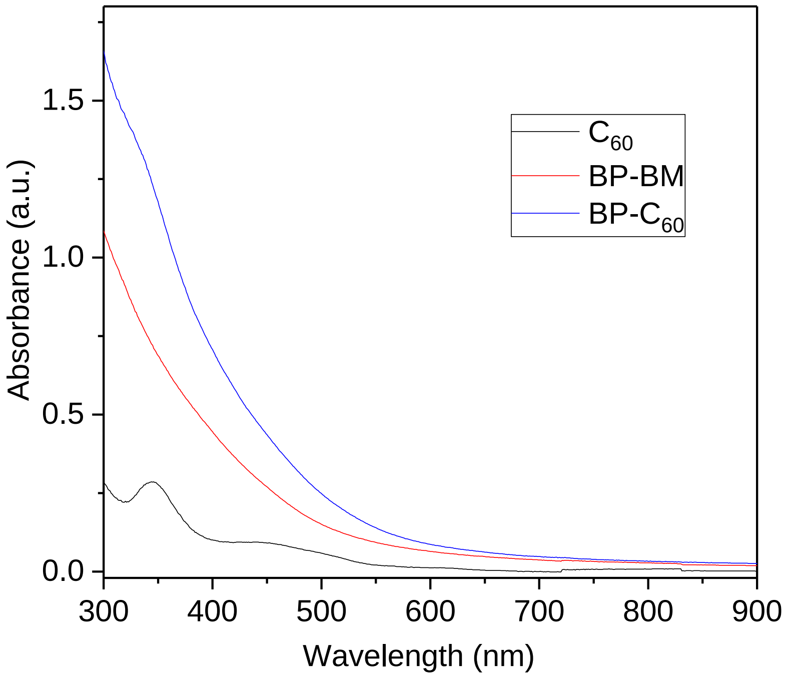

Supplementary Figure 11 | UV-vis-NIR absorption spectra of C_{60}, BP-BM and BP-C_{60} dispersed in water.

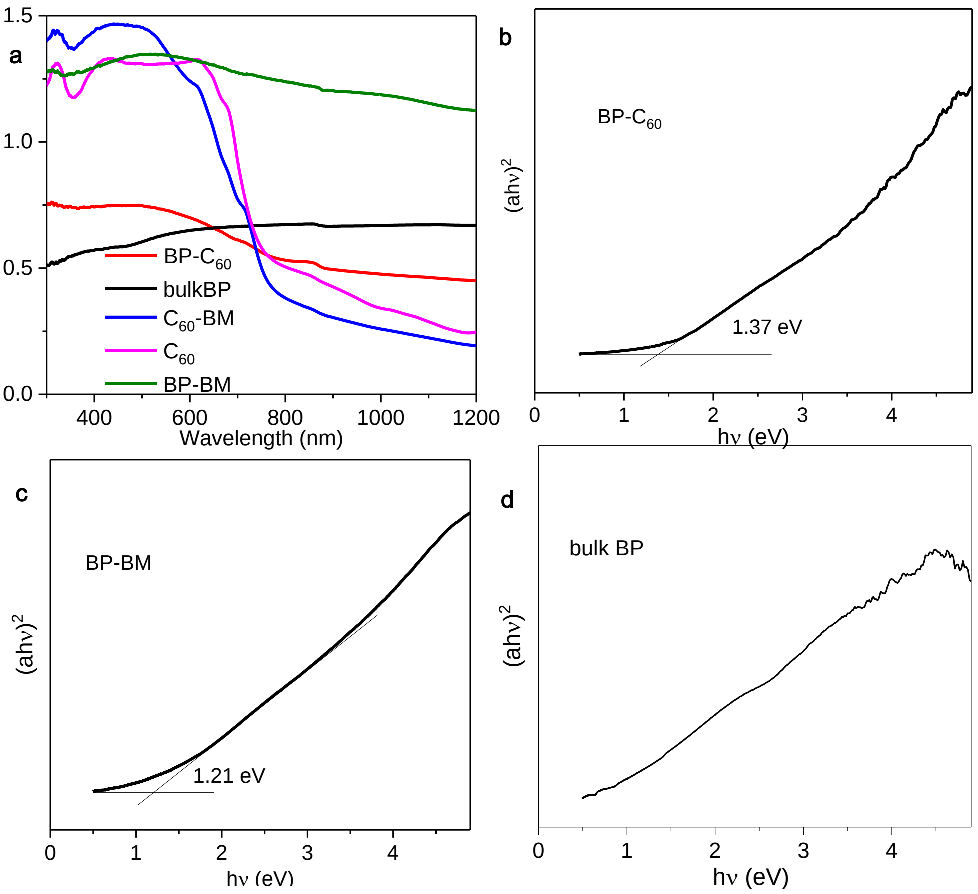

Supplementary Figure 12 | UV-vis diffuse reflectance spectra of the BP-C_{60} hybrid, BP bulk, C_{60}-BM, C_{60} and BP-BM, a, and $(ahv)^2$versus hv curve of the BP-C_{60} hybrid (b), BP-BM (c), and bulk BP (d). BP is a direct bandgap materials, thus r=2 for Tauc plot $(ahv)^r$ versus hv (ref. 1).

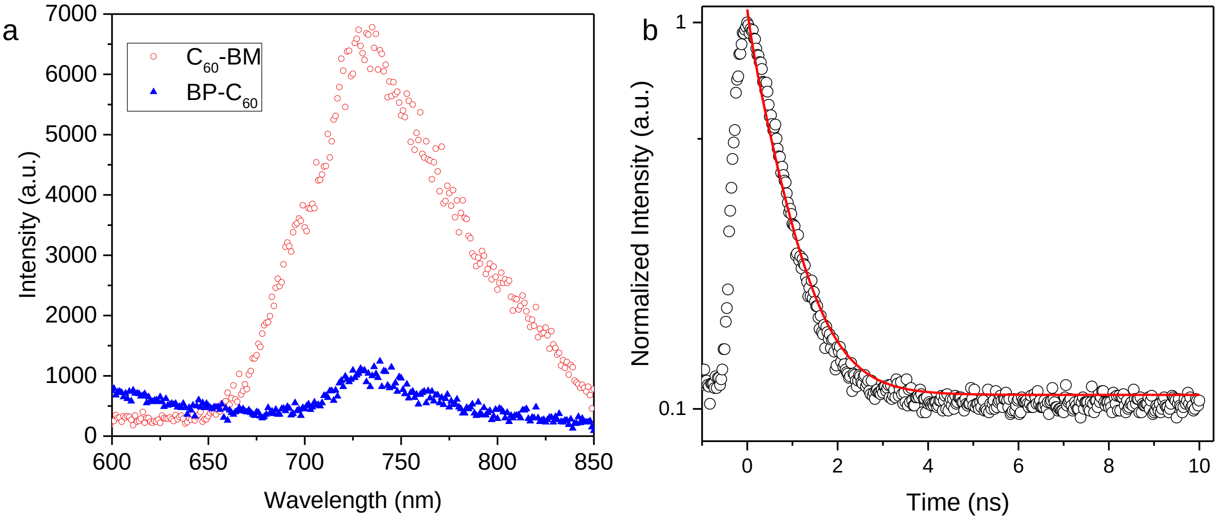

Supplementary Figure 13 | The steady-state and time-resolved photoluminescence (TRPL) spectrum. a, the steady-state photoluminescence spectra of C_{60}-BM and the BP-C_{60} hybrid (λ_{ex} = 450 nm). The PL intensity of C_{60}-BM is dramatically quenched in the spectrum of the BP-C_{60} hybrid, suggesting the photoinduced electron transfer from BP to C_{60} since the lowest unoccupied molecular orbital (LUMO) energy level of C_{60} (-4.5 eV *vs* energy level of vacumn (E_{vac})[3], converted to 0.0 V vs RHE) is much lower than that of the conduction band (CB) of BP-BM (-0.86 V vs RHE, see Figs. 4d and Table S1). The bulk BP and BP-BM show very weak PL signals (not shown), because their PL emissions are out of the detected range of our instrument. **b**, time-resolved photoluminescence (TRPL) spectrum (dotted curve) of the BP-C_{60} hybrid measured by a 532 nm laser (0.6 μW), which is fitted to a double exponential model (solid line). According to the fit with a double exponential model[4], the decay times attributed to trapping or defect-assisted recombination process (τ_d≈94 ps) and exciton radiative recombination (τ≈648 ps) respectively can be obtained.

a b

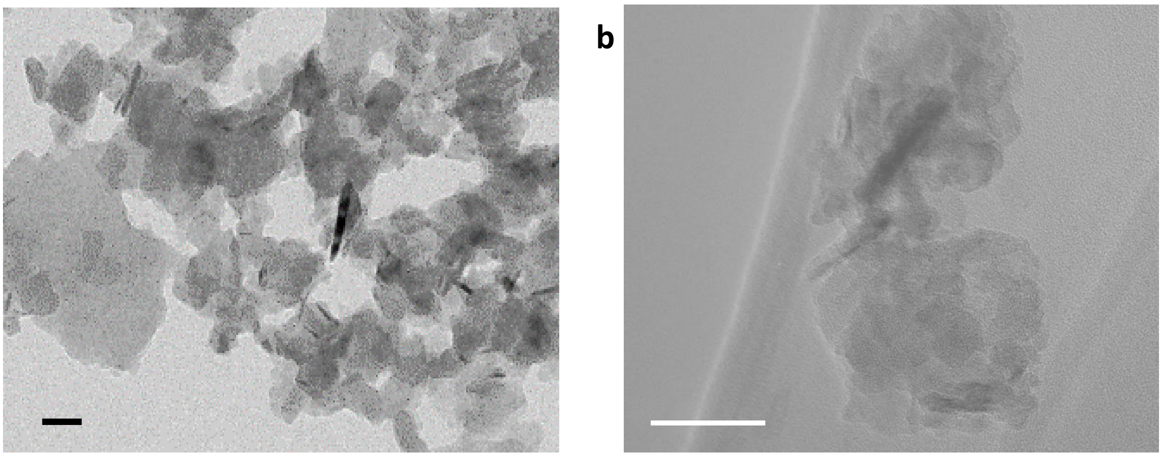

Supplementary Figure 14 | TEM image of the BP-C_{60} hybrid before and after photocatalytic degradation reactions. The scale bars are 50 nm.

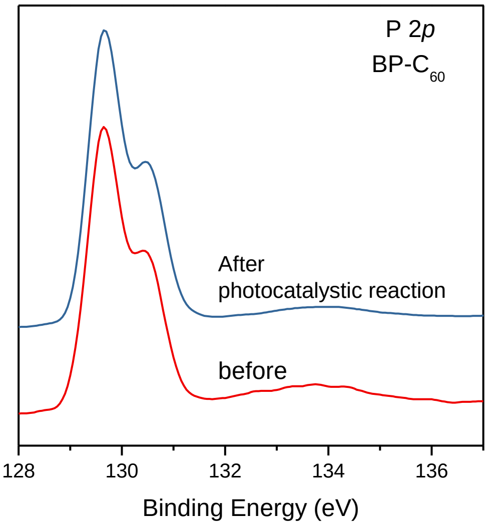

Supplementary Figure 15 | P 2*p* XPS spectrum of the BP-C_{60} hybrid before or after photocatalytic degradation reactions.

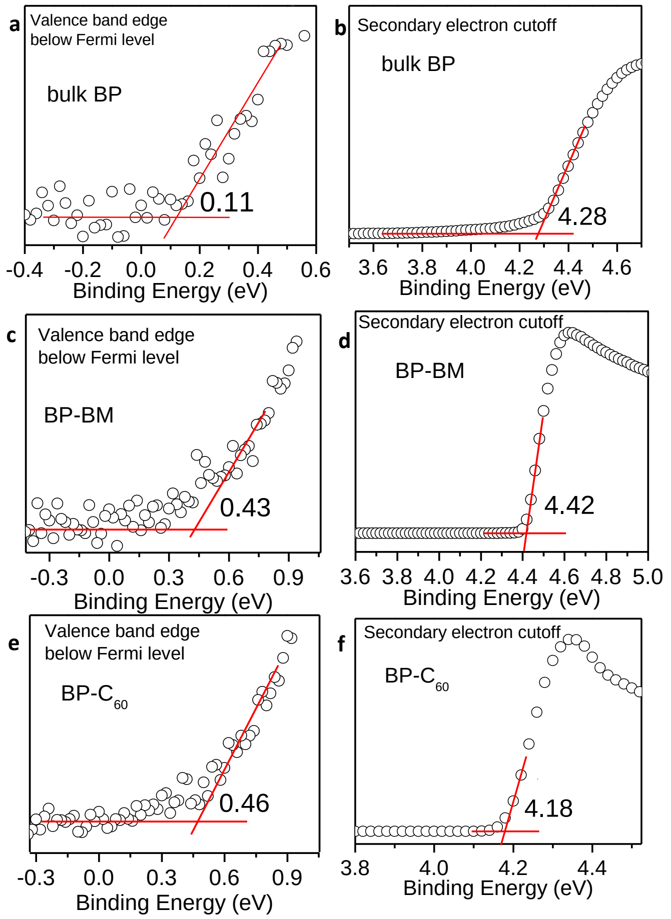

Supplementary Figure 16 | Synchrotron radiation photoemission spectra (SR-PES) of bulk BP (a, b) , BP-BM (c, d), and the BP-C_{60} hybrid (e, f). The left and right panels show the valance band edges and secondary electron cutoffs, respectively.

Supplementary Table 1. E_F, CB, VB energy levels and bandgaps of the BP-C_{60} bybrid, BP-BM and bulk BP.

Sample	Fermi Level (E_F, vs vacuum level) [a]	Fermi Level (E_F, vs RHE) [b]	Valance band maximum (E_{VB}, vs RHE) [c]	Conduction band minumum (E_{CB}, vs RHE) [d]	Bandgap (eV) [e]
BP-C_{60}	-4.18	-0.32	0.14	-1.23	1.37
BP-BM	-4.42	-0.08	0.35	-0.86	1.21
bulk BP	-4.28	-0.22	-0.11	-0.41	0.3 [f]

[a] Determined from the intersection point of the secondary electron cutoff shown in **Supplementary Fig. 16b, d, f**;

[b] According to conversion of energy level of vacumn (-4.5 eV) to energy level vs RHE (0.0 V)[3], E_F (vs RHE) = -4.5 - E_F (vs vacuum level);

[c] E_{VB} is calculated from valence band edge determined by SR-PES spectra shown in **Supplementary Fig. 16a, c, e**;

$E_{VB}=|\text{the value below fermi level}| + E_F$;

[d] E_{CB}= Bandgap - $|E_{VB}|$;

[e] Bandgap is estimated from $(\alpha h\nu)^2$ versus $h\nu$ curve shown in **Supplementary Fig. 12b, c**;

[f] The bandgap of bulk BP is from ref. 5.

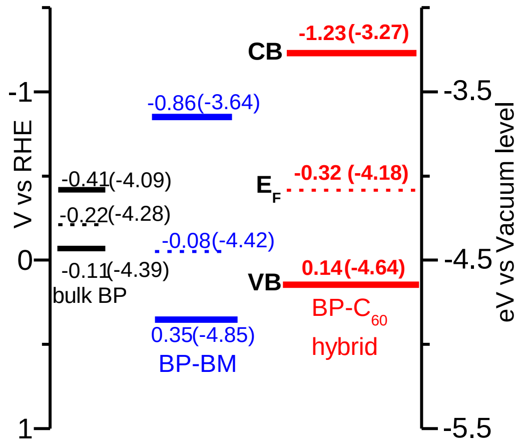

Supplementary Figure 17 | Energy level diagrams of bulk BP, BP-BM and BP-C_{60} hybrid based on the SR-PES and bandgap data. CB, VB, and E_F represent conduction band minumum, valance band maximum and Fermi level, respectively.

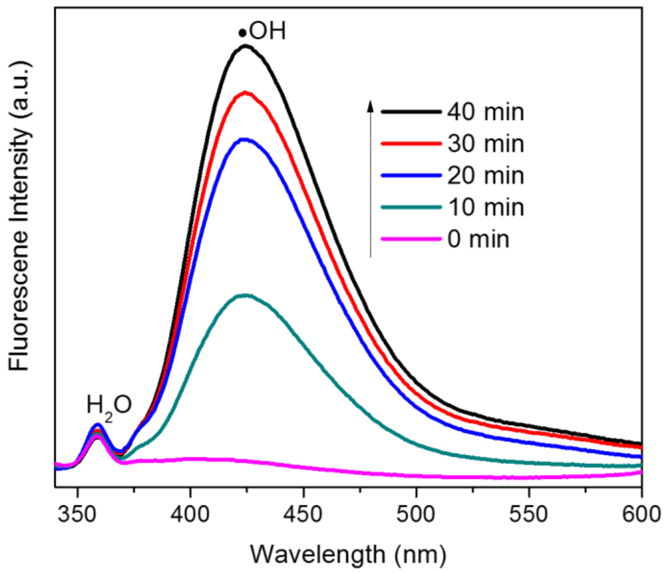

Supplementary Figure 18 | Time-dependent fluorescence spectra of terephthalic acid with the BP-C_{60} hybrid as photocatalyst (λ_{ex} = 325 nm). Typically, 5 mg BP-C_{60} hybrid powder was placed in 50 mL terephthalic acid (4 × 10^{-4} M) solution containing NaOH (2 × 10^{-3} M). The solution was irradiated by a 300 W halogen lamp (with a 400 nm filter). At every 10 min, 1.5 mL of the suspensions were collected and centrifuged. The resulted supernatants were subjected to PL measurements to detect the fluorescence spectra of the generated 2-hydroxyterephthalic acid.

Supplementary References

1. Zhu, X., *et al.* Black phosphorus revisited: a missing metal-free elemental photocatalyst for visible light hydrogen evolution. *Adv. Mater.* **29**, 1605776 (2017).
2. Hu, H., *et al.* Covalent functionalization of black phosphorus nanoflakes by carbon free radicals for durable air and water stability. *Nanoscale* **10**, 5834-5839 (2018).
3. Jeng, J.-Y. *et al.* $CH_3NH_3PbI_3$ Perovskite/Fullerene Planar-Heterojunction Hybrid Solar Cells, *Adv. Mater.* **25**, 3727-3732 (2013).
4. Surrente, A., *et al.* Onset of exciton-exciton annihilation in single-layer black phosphorus. *Phys. Rev. B* **94**, 075425 (2016).
5. Keyes, R. The electrical properties of black phosphorus. *Phys. Rev.* **92**, 580 (1953).
